# SAMHD1: a new contributor to HIV-1 restriction in resting CD4^+^ T-cells

**DOI:** 10.1186/1742-4690-9-88

**Published:** 2012-10-23

**Authors:** Li Wu

**Affiliations:** 1 Center for Retrovirus Research, Department of Veterinary Bioscience, Department of Microbial Infection and Immunity, The Ohio State University, 1900 Coffey Road, Columbus, OH, 43210, USA

## Abstract

Resting CD4^+^ T-cells are critical for establishing HIV-1 reservoirs. It has been known for over two decades that resting CD4^+^ T-cells are refractory to HIV-1 infection, but the underlying mechanisms are not fully understood. Compared with activated CD4^+^ T-cells that support HIV-1 infection, resting CD4^+^ T-cells have lower levels of dNTPs, which limit HIV-1 reverse transcription. The dNTPase SAMHD1 has been identified as an HIV-1 restriction factor in non-cycling myeloid cells. Two recent studies revealed that SAMHD1 restricts HIV-1 infection in resting CD4^+^ T-cells, suggesting a common mechanism of HIV-1 restriction in non-cycling cells that may contribute to viral immunopathogenesis.

## Introduction

HIV-1-induced CD4^+^ T-cell depletion and establishment of viral reservoir are two hallmarks during AIDS development. Resting CD4^+^ T-cells are critical for the establishment and maintenance of HIV-1 reservoirs through multifaceted mechanisms [1]. It has been known for over two decades that quiescent/resting CD4^+^ T-cells are highly refractory to HIV-1 infection [2,3], particularly to post-entry infection of R5-tropic, but not to X4-tropic viruses since resting CD4^+^ T-cells express the HIV-1 co-receptor CXCR4. However, the underlying mechanisms of HIV-1 restriction in resting CD4^+^ T-cells are not fully understood.

Compared with activated CD4^+^ T-cells that support productive HIV-1 infection, resting CD4^+^ T-cells have lower levels of dNTPs, which limit efficient HIV-1 reverse transcription and thereby block HIV-1 infection [3]. Addition of exogenous deoxynucleotides as dNTP precursors in quiescent CD4^+^ T-cells only partially rescues HIV-1 reverse transcription. The dNTP concentrations in quiescent CD4^+^ T-cells (0.3–5 μM) are approximately 6–10-fold lower relative to activated CD4^+^ T-cells (3–30 μM) and are around 6–100-fold higher compare with those in non-cycling macrophages (~0.05 μM) [3,4]. However, the molecular basis of maintaining low dNTP levels in non-cycling cells remained unclear until the recent discovery of a host protein named SAMHD1.

SAMHD1 was initially identified as an HIV-1 restriction factor in non-cycling myeloid cells, including primary monocytes, monocyte-derived dendritic cells and macrophages [5-7]. SAMHD1 has been proposed as a cellular regulator of the intrinsic antiviral response [8], while its physiological role remains largely unknown. As a dGTP-dependent deoxynucleotide triphosphohydrolase, SAMHD1 hydrolyzes intracellular dNTPs and reduces their concentrations to below the levels required for HIV-1 reverse transcription, thereby blocking viral infection in non-cycling myeloid cells [4]. These studies raised a significant question whether SAMHD1 contributes to HIV-1 restriction in resting CD4^+^ T-cells. If so, what are the implications of SAMHD1-mediated HIV-1 restriction in CD4^+^ T-cell depletion, establishment of the viral reservoir, and immune evasion?

### New findings and implications

**Figure 1:**
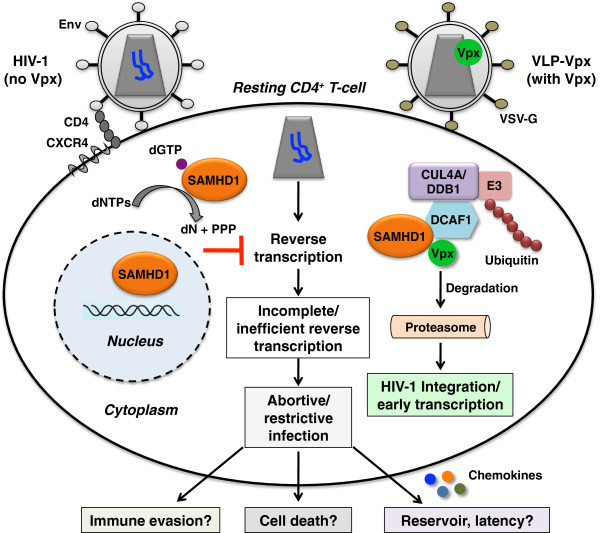
**SAMHD1 restricts HIV-1 infection in resting CD4**^**+**^**T-cells by limiting viral reverse transcription.** SAMHD1 is a dNTP triphosphohydrolase that converts intracellular dNTPs to deoxynucleosides (dN) and inorganic triphosphates (PPP), thereby limiting HIV-1 reverse transcription in resting CD4^+^ T-cells. Treatment of resting CD4^+^ T-cells with SIVsm/HIV-2 Vpx-containing VLPs or expression Vpx in resting CD4^+^ T-cells results in proteasomal degradation of SAMHD1 and leads to HIV-1 integration and early gene transcription. SAMHD1-mediated HIV-1 restriction in resting CD4^+^ T-cells might contribute to different aspects of viral immunopathogenesis, including CD4^+^ T-cell depletion and establishment of viral reservoirs and latency. Establishing HIV-1 latency in resting CD4^+^ T-cells requires chemokine-induced modifications in the actin cytoskeleton . Env, HIV-1 envelope protein.

Two recent studies revealed that SAMHD1 restricts HIV-1 infection in resting CD4^+^ T-cells [9,10], providing new insights into the mechanisms of HIV-1 restriction in non-cycling cells. SAMHD1-mediated HIV-1 restriction in myeloid cells has been proposed as an immune evasion strategy for HIV-1 to avoid antiviral innate and adaptive immune responses [5-7]. This proposed immune evasion mechanism likely also exists in resting CD4^+^ T-cells. In addition, HIV-1 restriction mediated by SAMHD1 in resting CD4^+^ T-cells may contribute to viral immunopathogenesis, such as T-cell depletion and establishment of the viral reservoir and latency (Figure 1).

Both studies by Baldauf *et al*. and Descours *et al*. demonstrated that resting CD4^+^ T-cells isolated from the peripheral blood of healthy donors express high levels of endogenous SAMHD1 protein, which are similar to those detected in primary myeloid cells or the monocytic THP-1 cell line [9,10]. Baldauf *et al*. showed that SAMHD1 is highly expressed in explants of human tonsil and likely localizes to non-proliferating macrophages, dendritic cells and CD4^+^ T-cells [9], which are the major target cell types when HIV-1 encounters lymphoid or mucosal tissues *in vivo*. Descours *et al*. further demonstrated that three circulating CD4^+^ T-cells subsets, including naïve (CD45RA^+^, CCR7^+^), central memory (CD45RA^-^, CCR7^+^) and effector memory cells (CD45RA^-^, CCR7^-^), express similarly high levels of SAMHD1 [10]. Moreover, circulating CD8^+^ T-cells and CD19^+^ B-cells also express SAMHD1 [10], although the physiologically relevant function of SAMHD1 and its regulation of dNTP levels in these cells remain to be examined. These results expanded our knowledge of SAMHD1 expression profile in major immune cell types that interact with HIV-1.

It has been shown that the lentiviral protein Vpx from HIV-2 and sooty mangabey-lineage SIV (SIVsm) counteracts SAMHD1-mediated HIV-1 restriction in myeloid cells through proteasomal degradation of SAMHD1 [5-7]. Similarly, Vpx from HIV-2 or SIVsm degrades SAMHD1 in resting CD4^+^ T-cells and efficiently increases single-cycle and replication-competent HIV-1 infection, which is correlated with increased products of full-length viral cDNA and 2-LTR circles in infected cells [9,10]. However, the degradation of SAMHD1 in resting CD4^+^ T-cells by treatment of Vpx-containing virus-like particles (VLP-Vpx) derived from SIV appears much less efficient compared with that in primary monocytes and activated CD4^+^ T-cells [10]. This might be due to distinct efficiencies of nuclear and cytoplasmic exchange among different cell types, given that Vpx-mediated SAMHD1 degradation is dependent on its nuclear export [11]. Furthermore, Vpx increases HIV-1 infection of all resting CD4^+^ T-cell subsets (naïve, central memory and effector memory cells) by approximately 8- to 15-fold [10]. Importantly, VLP-Vpx treatment neither induces CD4^+^ T-cell activation and proliferation nor affects HIV-1 infection of activated CD4^+^ T-cells [10], indicating that SAMHD1 specifically restricts HIV-1 infection in resting CD4^+^ T-cells, but not in activated CD4^+^ T-cells despite comparable expression levels of endogenous SAMHD1 in these cells.

Vpx-mediated SAMHD1 degradation in resting CD4^+^ T-cells can only enable early steps of HIV-1 life cycle, such as reverse transcription, but not later stages of HIV-1 replication [9,10]. Descours *et al*. found that, when SAMHD1 is degraded by Vpx in resting CD4^+^ T-cells, cytomegalovirus promoter-driven HIV-1 gene expression is significantly enhanced, while HIV-1 LTR promoter-driven viral gene expression remains blocked despite efficient reverse transcription [10]. These results suggest that HIV-1 transcriptional restriction in resting CD4^+^ T-cells is independent of SAMHD1. By contrast, Baldauf *et al*. showed that infection of resting CD4^+^ T-cells with a replication-competent HIV-1 carrying SIV Vpx significantly enhances viral gene expression, which is under the control of the HIV-1 LTR promoter [9]. Notably, Descours *et al*. used VSV-G-pseudotyped HIV-1 vectors in their infection assays [10], while Baldauf *et al*. used a CXCR4-tropic replication-competent HIV-1 [9]. Distinct envelope proteins of HIV-1 used in these studies and/or other different experimental conditions might result in the discrepancy of HIV-1 transcriptional gene expression, which remains to be confirmed. The use of VSV-G-pseudotyped HIV-1 vectors expands the host-cell range to beyond human CD4^+^ T-cells, suggesting that Descours *et al*. might examine SAMHD1 function in cell types that are not necessarily relevant to HIV-1 infection. Although Vpx treatment of resting CD4^+^ T-cells enables early steps of the HIV-1 and HIV-2 life cycle, it cannot enhance the production of HIV-1 p24 or HIV-2 p27 capsid proteins in the supernatants from the infected cells [9], suggesting that addition mechanisms are involved in HIV-1 and HIV-2 restriction in resting CD4^+^ T-cells.

Of note, T-cell activation does not affect SAMHD1 expression levels in CD4^+^ T-cells [9,10]. Consistent results were observed when CD4^+^ T-cell activation was achieved either with phytohemagglutinin treatment [9,10] or through T-cell receptor stimulation with anti-CD3/anti-CD28 in the presence of interleukin-2 [10]. Although activated CD4^+^ T-cells have 3- to 8-fold higher dATP/dTTP concentrations relative to resting CD4^+^ T-cells, SAMHD1 expression remains the same in resting and activated CD4^+^ T-cells [9]. It is likely that intracellular dNTP levels can be significantly increased when activated CD4^+^ T-cells become dividing cells. How activated CD4^+^ T-cells upregulate dNTP levels without down-regulating SAMHD1 expression remains to be investigated. Cellular ribonucleotide reductase increases the dNTP pool in cells through the *de novo* dNTP synthesis pathway. Thus, it is important to analyze expression levels and the activity of ribonucleotide reductases in resting and activated CD4^+^ T-cells to further understand the mechanisms by which SAMHD1 regulates the dNTP pool and HIV-1 infection efficiency in these cells.

SAMHD1 is primarily a nuclear protein [5,8] that has a nuclear localization signal in the N-terminus [11]. Using nuclear-cytoplasmic fractionation and three-dimensional reconstructions of confocal images, Baldauf *et al*. observed that large amounts of SAMHD1 in both the nucleus and cytoplasm of resting CD4^+^ T-cells, activated CD4^+^ T-cells, and macrophages [9], suggesting that SAMHD1 can be a cytoplasm-nucleus shuttling protein that depletes the dNTP pool in the cytoplasm of resting CD4^+^ T-cells and macrophages where HIV-1 reverse transcription happens. Further microscopy studies of SAMHD1 intracellular trafficking and its interactions with HIV-1 particles in resting CD4^+^ T-cells and myeloid cells will help dissect the molecular details of SAMHD1-mediated HIV-1 restriction.

Baldauf *et al*. showed that addition of exogenous deoxynucleotides in resting CD4^+^ T-cells or infection of resting CD4^+^ T-cells with Vpx-carrying HIV-1 increases the concentrations of intracellular dATP and dTTP, which correlates with enhanced HIV-1 infection [9]. These results suggest that the intracellular dNTP pools in resting CD4^+^ T-cells are rate limiting for HIV-1 reverse transcription, and SAMHD1 plays a regulatory role in this process. Furthermore, silencing SAMHD1 expression in post-activated resting CD4^+^ T-cells using specific siRNA or shRNA significantly increases HIV-1 infection, further suggesting that SAMHD1 suppresses HIV-1 infection in post-activated resting CD4^+^ T-cells [9].

*SAMHD1* gene mutations are associated with Aicardi-Goutières syndrome (AGS), an inherited autoimmune disease mimicking congenital viral infection with elevated type I interferon production [8]. Berger *et al*. have reported that, within peripheral blood mononuclear cells (PBMCs) from SAMHD1-deficient AGS patients, CD14^+^ monocytes are more susceptible to HIV-1 infection compared with cells from healthy donors [7]. Baldauf *et al*. and Descours *et al*. further demonstrated that HIV-1 infection of SAMHD1-deficient resting CD4^+^ T-cells in PBMCs from AGS patients is significantly increased compared with that of SAMHD1-expressing normal cells [9,10]. Moreover, VLP-Vpx treatment of SAMHD1-deficient resting CD4^+^ T-cells cannot further enhance HIV-1 infection [10], indicating that SAMHD1 is necessary for Vpx to relieve HIV-1 restriction in resting CD4^+^ T-cells. These *ex vivo* studies using SAMHD1-deficient cells provided further evidence that SAMHD1 functions as an HIV-1 restriction factor.

It is conceivable that SAMHD1-mediated HIV-1 restriction in non-cycling cells may play an important role in viral immunopathogenesis. Doitsh *et al*. found that abortive HIV-1 reverse transcription in resting tonsil CD4^+^ T-cells results in cell death through proapoptotic and proinflammatory response, which is triggered by accumulation of incomplete HIV-1 reverse transcripts [12]. However, it is unclear whether and how SAMHD1-mediated restriction of HIV-1 in resting CD4^+^ T-cells may trigger innate immune recognition of viral cDNA and result in cell death.

## Conclusions and future directions

The studies by Baldauf *et al*. and Descours *et al*. revealed that SAMHD1 restricts HIV-1 infection in resting CD4^+^ T-cells by limiting viral cDNA synthesis via depleting the intracellular dNTP pool, at least in part [9,10]. SAMHD1-mediated HIV-1 restriction in myeloid cells and resting CD4^+^ T-cells is likely a general mechanism to protect host cells from productive viral infection, which can also be a potential strategy of HIV-1 immune evasion to avoid efficient anti-viral innate immunity [13].

Because addition of deoxynucleotides in resting CD4^+^ T-cells only partially restores HIV-1 reverse transcription, other mechanisms may also contribute to HIV-1 restriction imposed by SAMHD1 in non-cycling cells. It is possible that SAMHD1 requires a cellular cofactor in non-cycling cells for its HIV-1 restriction function. On the contrary, it is also plausible that T-cell activation or myeloid cell differentiation diminishes the expression and/or function of a SAMHD1 suppressor, thereby rendering HIV-1 restriction activity of SAMHD1. Further studies are needed to delineate the cellular mechanism by which SAMHD1 inhibits HIV-1 infection in non-cycling cells.

We have reported that polymorphisms of the *SAMHD1* gene are not associated with HIV-1 infection and natural control in Europeans and African-Americans [14]. It would be important to examine the role of SAMHD1 in HIV-2 infection given that HIV-1 has not evolved a viral antagonist to SAMHD1. It is also important to further study the effect of Vpx on SIV infection and viral immunopathogenesis using SIV and non-human primate models. *SAMHD1* gene knockout mice are currently unavailable. It would be intriguing to examine whether *SAMHD1* knockout mice mirror the AGS symptoms in human patients and become more susceptible to the infection with murine retroviruses or other RNA or DNA viruses. The *SAMHD1* knockout mice will also be valuable to understand the role SAMHD1 in innate and adaptive immunity.

Post-transcriptional regulation and/or post-translational modifications of SAMHD1 might be important for its function in restricting HIV-1 infection in non-cycling cells. For example, a new study by Welbourn *et al*. identified naturally occurring splice variants of SAMHD1 in a variety of cell types [15]. These splice variants do not have the dNTPase activity and they are metabolically unstable and therefore can be rapidly degraded in the absence of Vpx [15], suggesting a post-transcriptional mechanism regulating the hydrolase activity of SAMHD1.

Further elucidating the mechanisms of SAMHD1-mediated HIV-1 restriction in non-cycling myeloid cells and resting CD4^+^ T-cells may help develop new therapeutic approaches against HIV-1 infection. For instance, one can use pharmacologic induction of low dNTP pools in HIV-1 target cells to inhibit viral infection or to block SAMHD1 activity to elicit protective anti-HIV-1 immune responses. However, these strategies likely will be used in combination with other available anti-HIV-1 interventions and need to be carefully designed and evaluated to avoid potential detrimental effects on the host.

## Abbreviations

SAMHD1: Sterile alpha motif domain- and HD domain-containing protein 1; HIV-1: Human immunodeficiency virus type 1; HIV-2: Human immunodeficiency virus type 2; SIV: Simian immunodeficiency virus; dNTP: Deoxynucleoside triphosphate; dNTPase: dNTP triphosphohydrolase; VSV-G: Vesicular stomatitis virus G protein; LTR: Long terminal repeat; siRNA: Small interfering RNA; shRNA: Short hairpin RNA.

## Competing interests

The author declares that he has no competing interests.

## Authors’ contributions

LW conceived the topic and wrote the manuscript.

## References

[B1] Eisele E Siliciano RF Redefining the Viral Reservoirs that Prevent HIV-1 Eradication Immunity 2012 37 3 377 388 10.1016/j.immuni.2012.08.010 22999944 PMC3963158

[B2] Zack JA Arrigo SJ Weitsman SR Go AS Haislip A Chen IS HIV-1 entry into quiescent primary lymphocytes: molecular analysis reveals a labile, latent viral structure Cell 1990 61 2 213 222 10.1016/0092-8674(90)90802-L 2331748

[B3] Gao WY Cara A Gallo RC Lori F Low levels of deoxynucleotides in peripheral blood lymphocytes: a strategy to inhibit human immunodeficiency virus type 1 replication Proc Natl Acad Sci U S A 1993 90 19 8925 8928 10.1073/pnas.90.19.8925 7692440 PMC47473

[B4] Lahouassa H Daddacha W Hofmann H Ayinde D Logue EC Dragin L Bloch N Maudet C Bertrand M Gramberg T SAMHD1 restricts the replication of human immunodeficiency virus type 1 by depleting the intracellular pool of deoxynucleoside triphosphates Nat Immunol 2012 13 3 223 228 10.1038/ni.2236 22327569 PMC3771401

[B5] Laguette N Sobhian B Casartelli N Ringeard M Chable-Bessia C Segeral E Yatim A Emiliani S Schwartz O Benkirane M SAMHD1 is the dendritic- and myeloid-cell-specific HIV-1 restriction factor counteracted by Vpx Nature 2011 474 7353 654 657 10.1038/nature10117 21613998 PMC3595993

[B6] Hrecka K Hao C Gierszewska M Swanson SK Kesik-Brodacka M Srivastava S Florens L Washburn MP Skowronski J Vpx relieves inhibition of HIV-1 infection of macrophages mediated by the SAMHD1 protein Nature 2011 474 7353 658 661 10.1038/nature10195 21720370 PMC3179858

[B7] Berger A Sommer AF Zwarg J Hamdorf M Welzel K Esly N Panitz S Reuter A Ramos I Jatiani A SAMHD1-deficient CD14+ cells from individuals with Aicardi-Goutieres syndrome are highly susceptible to HIV-1 infection PLoS Pathog 2011 7 12 e1002425 10.1371/journal.ppat.1002425 22174685 PMC3234228

[B8] Rice GI Bond J Asipu A Brunette RL Manfield IW Carr IM Fuller JC Jackson RM Lamb T Briggs TA Mutations involved in Aicardi-Goutieres syndrome implicate SAMHD1 as regulator of the innate immune response Nat Genet 2009 41 7 829 832 10.1038/ng.373 19525956 PMC4154505

[B9] Baldauf HM Pan X Erikson E Schmidt S Daddacha W Burggraf M Schenkova K Ambiel I Wabnitz G Gramberg T SAMHD1 restricts HIV-1 infection in resting CD4(+) T cells Nat Med 2012 Epub ahead of print 10.1038/nm.2964 PMC3828732 22972397

[B10] Descours B Cribier A Chable-Bessia C Ayinde D Rice G Crow Y Yatim A Schawartz O Laguette N Benkirane M SAMHD1 restricts HIV-1 reverse transcription in quiescent CD4+ T-cells Retrovirology 2012 9 1 87 Epub in Oct 2012 10.1186/1742-4690-9-87 23092122 PMC3494655

[B11] Brandariz-Nunez A Valle-Casuso JC White TE Laguette N Benkirane M Brojatsch J Diaz-Griffero F Role of SAMHD1 nuclear localization in restriction of HIV-1 and SIVmac Retrovirology 2012 9 49 10.1186/1742-4690-9-49 22691373 PMC3410799

[B12] Doitsh G Cavrois M Lassen KG Zepeda O Yang Z Santiago ML Hebbeler AM Greene WC Abortive HIV infection mediates CD4 T cell depletion and inflammation in human lymphoid tissue Cell 2010 143 5 789 801 10.1016/j.cell.2010.11.001 21111238 PMC3026834

[B13] St Gelais C Wu L SAMHD1: a new insight into HIV-1 restriction in myeloid cells Retrovirology 2011 8 1 55 10.1186/1742-4690-8-55 21740548 PMC3142215

[B14] Coon S Wang D Wu L Polymorphisms of the SAMHD1 Gene Are Not Associated with the Infection and Natural Control of HIV Type 1 in Europeans and African-Americans AIDS Res Hum Retroviruses 2012 Epub on April 26 10.1089/aid.2012.0039 PMC3505062 22530776

[B15] Welbourn S Miyagi E White TE Diaz-Griffero F Strebel K Identification and characterization of naturally occurring splice variants of SAMHD1 Retrovirology 2012 9 1 86 Epub in Oct 2012 10.1186/1742-4690-9-86 23092512 PMC3503569

